# Therapeutic potential of magnesium sulfate in improving duodenal homeobox 1 and PPARG coactivator 1 alpha genes to reduce pancreatic insulin resistance in F1 offspring of diabetic rats

**DOI:** 10.22038/ijbms.2025.84255.18232

**Published:** 2025

**Authors:** Mahtab Ghanbari Rad, Hossein Rezazadeh, Mohammadreza Sharifi, Nepton Soltani

**Affiliations:** 1 Department of Physiology, School of Medicine, Isfahan University of Medical Sciences, Isfahan, Iran; 2 Gerash Cellular and Molecular Research Center, Gerash University of Medical Sciences, Gerash, Iran; 3 Department of Genetics and Molecular Biology, School of Medicine, Isfahan University of Medical Sciences, Isfahan, Iran; 4 Applied Physiology Research Center, Cardiovascular Research Institute, Isfahan University of Medical Sciences, Isfahan, Iran

**Keywords:** Diabetes, Euglycemic - hyperinsulinemic clamp, Insulin resistance, Magnesium, Pancreas

## Abstract

**Objective(s):**

The study aimed to investigate the role of magnesium sulfate (MgSO_4_) therapy in pancreatic insulin resistance (IR) in diabetic and non-diabetic rats and their F1 offspring following administration of a high-fat diet (HFD).

**Materials and Methods:**

Diabetes was induced in the subjects through a combination of HFD and a low dose of streptozotocin (STZ). The male and female diabetic animals were divided into three groups: diabetic control (DC), insulin, and MgSO_4_ (Mg) treated groups. One group of both sexes was kept as non-diabetic control (NDC) and fed a regular diet. Their F1 offspring were fed a regular diet for four months. Euglycemic hyperinsulinemic clamp (HEC) tests were performed on the parents and their F1 offspring. Blood samples were taken every hour during the clamp to measure changes in glucagon levels. Pancreas tissue was isolated, and the expression of pancreatic and duodenal homeobox 1(Pdx1) and PPARG coactivator 1 alpha (Pgc1α) genes was measured in all groups.

**Results:**

Treatment with MgSO_4_ or insulin decreased HOMA-IR, TyG index, BGL, ITT, HbA1c, glucagon level, Pgc1α expression, and increased glucose infusion rate (GIR), body weight, Pdx1 gene expression, and insulin level in diabetic parents and their F1 offspring compared to the DC group. These changes suggest a decrease in IR. Additionally, alterations in IR have decreased. Also, changes in the expression of these genes indicate a positive impact on the survival and regeneration rate of pancreatic β cells.

**Conclusion:**

MgSO_4_ showed beneficial effects in treating glucose metabolism and improving IR.

## Introduction

Diabetes mellitus (DM) is characterized by persistent hyperglycemia due to impaired insulin secretion, resistance to insulin’s peripheral actions, or both. In type 2 diabetes (T2DM), the insulin response is reduced, which is defined as insulin resistance (IR) (1). 

Initially, insulin production is increased by pancreatic β-cells to prevent obesity-related IR (2). However, diabetes and β-cell failure that occur after this time may be caused by an insufficient proliferation of the β-cell mass or resistance of the β-cell mass to glucose (3). Under normal conditions, insulin suppresses glucagon secretion from pancreatic α-cells. However, when IR is present in diabetic pancreatic α-cells, insulin can no longer suppress glucagon secretion from α-cells, leading to oversecretion. IR in pancreatic α-cells means a state in which α-cell insulin signaling is weakened (4). As a result, hyperinsulinemia serves as an effective compensating mechanism to maintain insulin activity in IR (5). Fat accumulation outside the subcutaneous adipose tissue often adversely affects systemic metabolism. Increased FFAs cause mitochondrial dysfunction and lipotoxicity (6). Chronic accumulation of fat in the pancreas can impair glucose metabolism and insulin secretion (7). Placental enzymes produce fatty acids from maternal triglycerides and circulating phospholipids as fatty acids migrate through the placenta (8).

The maintenance and survival of pancreatic cells greatly depend on homeobox 1 and duodenal transcription factor (Pdx1) (9). In diabetes, reduced *Pdx1* expression contributes to β-cell loss and dysfunction. Therefore, boosting *Pdx1* expression may be a successful tactic for maintaining β-cell mass and function. (10). 

Glucose intolerance and mitochondrial dysfunction are impacted by the peroxisome proliferator-activated receptor gamma coactivator alpha (Pgc-1 α). Dysfunction in β-cells has also been connected to *Pgc-1α* overexpression. *Pgc-1 α* overexpression significantly reduces the amount of glucose-dependent insulin secretion (11).

 The function of insulin produced by pancreatic β-cells and glucose homeostasis are both significantly influenced by magnesium (Mg^+2^). In T2DM, low Mg^+2^ concentrations induce peripheral IR (12). Previous studies have indicated that MgSO_4_ reduced IR in male T2DM rats. Additionally, giving MgSO_4_ to parents with T2DM reduced muscle IR in both parents and their F1 offspring (13). Mg^+2^ deficiency leads to decreased insulin sensitivity and impaired glucose tolerance(13, 14), while higher Mg^+2^ levels are associated with a reduced risk of T2DM (15). According to a study, patients with T2DM who received Mg^+2^ supplements had better blood glucose control indicators (16). Research indicates fatty acids can migrate across the placenta, which is crucial for various cellular processes in the developing fetus (17, 18). Additionally, the placenta’s enzymes assist in converting maternal triglycerides and circulating phospholipids into fatty acids (17). The transfer of fatty acids from the placenta to the infant can negatively affect infant glucose metabolism and insulin sensitivity (19). Given the importance of MgSO_4_ in treating diabetes and reducing IR, investigating whether MgSO_4_ consumption in diabetic parents can affect reducing IR and improving the metabolic status (20-22) of their first-generation infants could also be helpful for broad future insights.

Therefore, the present study investigated the effects of MgSO_4_ on glucose, insulin, glucagon levels, and IR index in male and female rats. These rats had parents who were made diabetic with streptozotocin (STZ) injections and received a high-fat diet (HFD). The HEC method was used to investigate IR. In this study, we examined IR in both male and female rats and their F1 offspring and the role of MgSO_4_ in improving IR using the euglycemic-hyperinsulinemic clamp method in conscious rats. Additionally, we explored the expressions of the *Pdx1 and* *Pgc1α* genes in these animals. We also investigated whether treating diabetic parents affected the development of diabetes in their F1 offspring. Due to cost limitations, we did not investigate IR in pancreatic β and α cell lines, focusing instead on measuring this index peripherally. We chose to work with animals to observe if the administration of MgSO_4_ could reduce IR at the pancreas level in an animal’s model of T2DM and their F1 offspring. Previous studies have shown that MgSO_4_ administration reduces IR in muscle.

## Materials and Methods

### Animals

Wistar rats that were 4–5 weeks old and weighed between 120 and 150 g at birth were utilized in this study. The animal work was conducted in the endocrine laboratory of Isfahan University of Medical Sciences, and our animal ethics license number was IR.MUI.MED.REC.1399.1070. All procedures were carried out by the necessary regulations and guidelines. The animals were housed at a controlled room temperature of 22±2 °C and relative humidity of 50±5% with a light/dark cycle of 12:12 hours. Each cage housed a similar number of rats in all groups. The rats were provided with a pellet diet and unrestricted water access. 

In this study, efforts were made to minimize animal stress using noninvasive methods such as administering magnesium sulfate via drinking water. Animals were kept in controlled environments, and their health was regularly monitored. Any signs of stress or health issues led to immediate removal from the study to prevent further suffering.

### Diabetes induction and study design

Three months of a high-fat diet (HFD) (Table 1) were administered to the rats, followed by an intraperitoneal injection of 35 mg/kg of streptozotocin (STZ) to induce T2DM. Blood glucose levels (BGL) were measured using a glucometer (Ascensia Elite XL, Germany) via the tail vein (23, 24). Rats with BGL above 250 mg/dl were considered to have T2DM. The animals were randomly divided into four groups (n=7): the non-diabetic control (NDC) group, which received a standard diet throughout the experiment; the diabetic control group (DC), which received HFD and STZ but no treatment; the insulin-treated group (Ins), where diabetic rats received HFD and 2.5 u/kg insulin (Elixir, Iran) twice daily intraperitoneally (IP), and a diabetic group treated with 10 g/l of MgSO_4_ (Sigma-Aldrich, Hamburg, Germany) in their drinking water (Mg). Throughout the treatment, the animal’s body weight (measured weekly using a digital scale in France) and BGL were monitored (25). All female and male rats were fed an HFD d during mating, pregnancy, and breastfeeding. The F1 offspring were then given a regular diet and entered the treatment study at 4 weeks of age, continuing for 4 months.

In this study, the number of male and female rats in each group was as follows: In the parental groups, there were 28 males and 28 females, and in the F1 offspring group, there were 28 males and 28 females.

### Insulin tolerance test (ITT)

Animals from all groups underwent ITT in the latest month. All animals were administrated 2.5 U/kg of insulin through an IP injection before blood was drawn from the tail vein. Following the insulin administration, glucose levels were measured at 0, 20, 30, 40, 60, 90, and 120 min (9). This test was conducted for both parents 3 months after the treatment and for the cubs at the end of the fourth month.

### Mating, childbirth, and lactation

After two months of HFD treatment, male and female rats of all four groups were placed in separate cages for mating (without genetic mixing); after birth, the males were separated. Female animals during pregnancy and lactation were kept in the same conditions as their respective groups. After delivery and breastfeeding, both parents underwent a clamp test, and blood samples were collected.

### Euglycemic hyperinsulinemic clamp

Rats were sedated with ketamine (100 mg/kg, IP, Rotexmedica, Trittau, Germany) and xylazine (8 mg/kg, IP) at the end of the experiment. The left common carotid artery and right jugular vein were cannulated with heparinized polyethylene. The cannulas were transferred to the animal’s back and fixed in place. The rats were returned to their cages and allowed to recover after three to five days. During the postoperative recovery, 150 U/ml of heparinized saline was injected into both tubes. Daily, once the rats had fully recovered, they were weighed after fasting overnight. A microinjection pump was connected to administer insulin at a constant rate of 20 mu/kg/min and glucose at a variable rate of 25%. Blood samples were taken every 5 to 10 min to measure BGL throughout the 5-hour experiment for each rat. Blood samples were collected every hour to measure glucagon levels during the clamp, and final samples were taken to measure insulin levels. BGL was maintained at 100 ± 5 mg/dl for the last 30 min of the clamp period in rats. Finally, the glucose infusion rate (GIR) was used to assess insulin-stimulated glucose uptake during the final 30 min of the clamp and whole-body IR (25).

### HbA1c measurement

Using commercially available kits (Pishtaz Tab, Tehran) and a Hitachi 717 autoanalyzer (Roche Diagnostics, Basel, Switzerland), HbA1c was assessed in accordance with the manufacturer’s instructions (26). 

### Pancreatic tissue preparation

After completing the euglycemic-hyperinsulinemic clamp, the animals were anesthetized with a high dose of ketamine and xylazine (IP). They were then sacrificed, and their pancreatic tissue was isolated. After washing in cold isotonic saline, 50–100 mg of tissue was placed in a DNase/RNase-free microtube and stored at -80 °C to preserve the expression of the *Pgc1α* and *Pdx1* genes (9).

### Real-time PCR

After extraction of total RNA using the Trizol kit (RNA Anacell, Iran), 5-10 ng of total RNA was used for the synthesis of complementary DNAs (cDNA) using M-MLV RT (Anacell, lot N: CS0021) according to the manufacturer’s instructions. Expression of mRNA was measured using quantitative real-time PCR (qPCR) with SYBR-green in a stage I real-time PCR machine from Applied Biosystems. 1µ of total cDNA was mixed with ten microliters of 2x SYBR Green PCR mixed with ROX, treated water, and 10 pmol/ml of each sense and antisense primers for the measured genes. Table 2 shows the primers used in this study. The CT method was utilized to determine the relative expression levels, with the average expression of beta-actin serving as the internal reference gene (27) (11). 

### Biomedical assay design

Plasma insulin and glucagon levels were assessed by ELISA kits (Zell Bio GmbH, Germany) according to the manufacturer’s instructions. HOMA-IR and TyG indices were also calculated based on the following formulas.

TyG: (*Fasting blood glucose levels* (mg/dl)*TG (mg/dl))/2 (28).

HOMA-IR :( *fasting glucose* * *fasting insulin*)/22.5 (29)

### Statistical analysis

Data were reported as mean ± SEM. One-way and two-way analyses of variance, as well as Tukey’s *post hoc* test, were used to compare groups. Statistical significance was defined as a *P*-value of less than 0.05 (*P*<0.05). SPSS software was used for all statistical analyses.

## Results

### Effect of MgSO4 on blood glucose levels (BGL)

**Figure 1:**
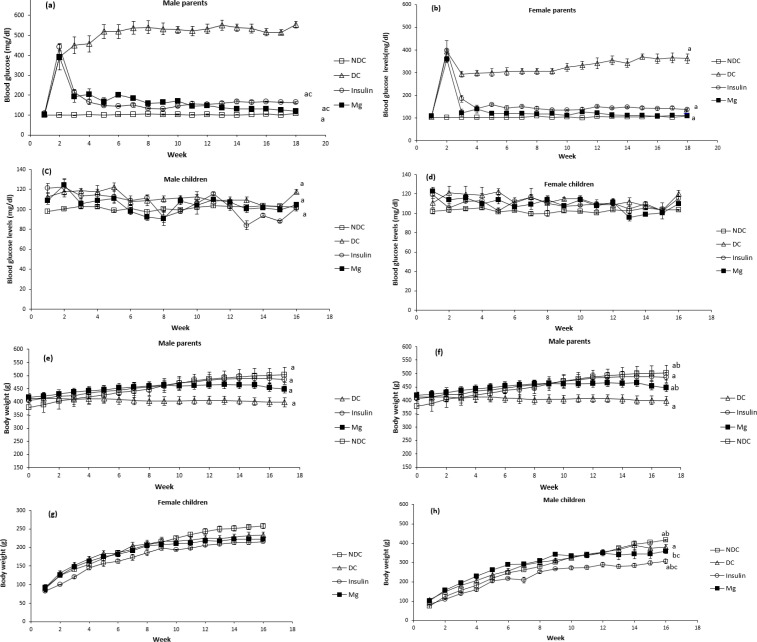
Comparison of blood glucose levels (BGL) s in male parents (a), female parents (b), male pups (c), female pups (d) and body weight of animals in male parents (e), female parents (f), male pups (g), female pups (h) in non-diabetic control group (NDC) were fed standard diet, diabetic control group received high-fat diet and 35 mg/kg STZ (DC), diabetic animals were treated with 10 g/l MgSO_4_ by drinking water (Mg) and diabetic animals were treated with insulin (2.5 U/kg twice per day) (Insulin). STZ: Streptozotocin

BGL was measured weekly in all groups. The induction of diabetes in male and female animals led to a significant increase (*P*<0.0001) in BGL. In comparison to the NDC group, BGL remained elevated in the DC group for 17 weeks. After receiving MgSO_4_ or insulin injection for 17 weeks, it was observed that MgSO_4_ significantly reduced (*P*<0.0001) plasma glucose concentration in both sexes compared to the DC group. Therefore, our findings demonstrate that MgSO_4_ or insulin therapy significantly reduced BGL in both males and females (Figure 1a-d). No significant changes in weekly BGL were observed in any of the groups.

Our results showed that the blood glucose level in the F1 offspring of DC group parents was significantly (*P*<0.001) higher than that of the F1 offspring of NDC group parents. Also, in the F1 offspring of parents treated with MgSO_4_ and insulin, blood glucose level was significantly (*P*<0.001) lower than that of the F1 offspring of diabetic parents.

Parents: NDC male: 107 ± 1.3 mg/dl vs DC male: 553.5 ± 17.7 mg/dl; NDC female: 109.6± .5 mg/dl vs DC female: 362.6 ± 20.7mg/dl, Mg male: 119 ± 3.09 mg/dl; Mg female: 111.4 ± 1.6 mg/dl; insulin male: 163 ± 3.9mg/dl; insulin female: 136.5 ± 6.2 mg/dl, *P*<0.0001).

Our findings also showed that the body weight in the DC group was significantly reduced (*P*<0.001) compared to the NDC group. Additionally, in the treatment groups receiving insulin or MgSO_4_, there was a significant increase (*P*<0.0001) compared to the DC group (Figure 1e-h).

### Effect of MgSO4 on insulin tolerance test (ITT)

**Figure 2:**
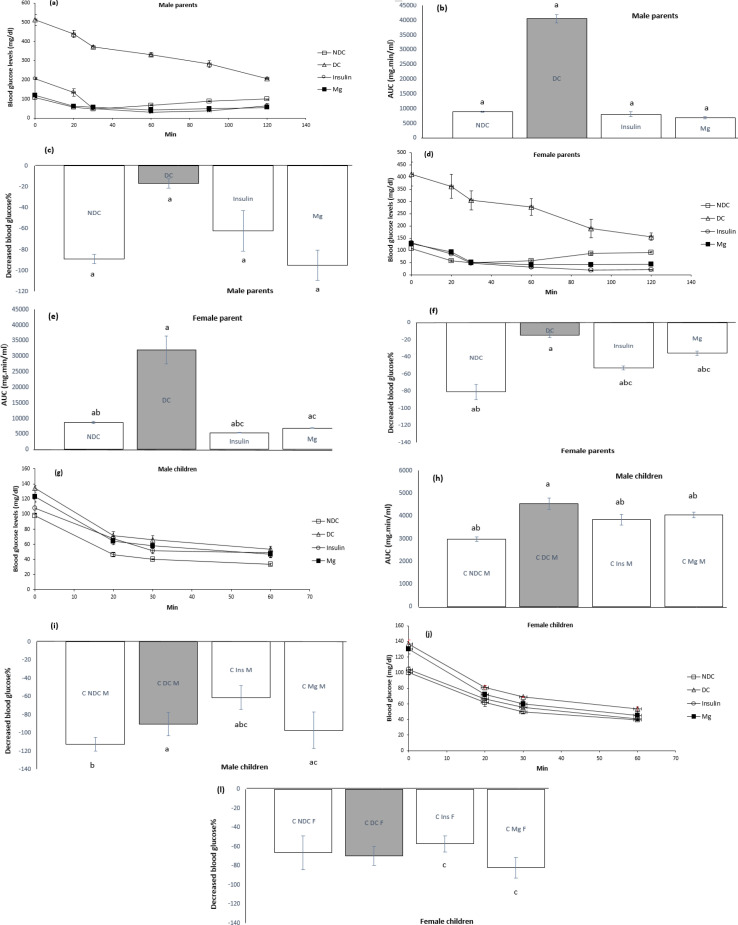
Comparison of insulin tolerance test (ITT) in male parents (a), female parents (d), male pups (g), female pups (j), the area under the glycemic curve (AUC) for male parents (b), female parents (e), male pups (h), female pups (k) and their decreased BGL in male parents (c), female parents (f), male pups (i), female pups (l) in the non-diabetic control group (NDC) were fed standard diet, diabetic control group received high-fat diet and 35 mg/kg STZ (DC), diabetic animals were treated with 10 g/l MgSO_4_ by drinking water (Mg) and diabetic animals were treated with insulin (2.5 U/kg twice per day) (Insulin).

In the last month of treatment, an ITT was performed on all groups of parents and their F1 offspring. Our results showed that the area under the curve (AUC) in the ITT was significantly increased (*P*<0.0001) in the DC group in both male and female parents and their F1 offspring compared to the NDC group. The AUC in groups receiving MgSO_4_ and insulin significantly decreased (*P*<0.001) compared to the DC group in both sexes. The percentage of reduction in BGL was also higher in the female parents receiving MgSO_4_ compared to the DC group. The results of this test in both sexes of F1 offspring were that the diabetic group (DC) showed a significantly higher AUC (*P*<0.001) compared to the NDC group, and the AUC in the groups treated with MgSO4 and insulin compared to the group DC decreased (*P*<0.01), (Figure 2a-l).

### Effect of MgSO4 on glucose infusion rate (GIR)

HEC was performed in all male and female animals to evaluate the IR of the whole body at the end of the treatment. The BGL was clamped at 100 ± 5 mg/dl in this test, and the GIR factor was calculated. GIR was significantly decreased (*P*<0.0001) in male and female DC groups compared to male and female NDC groups. Administration of insulin or MgSO_4_ significantly increased (*P*<0.001) GIR in both sexes compared to the DC group. Additionally, our findings showed that administration of MgSO_4_ is more effective in reducing body IR than insulin. MgSO_4_ or insulin, while able to reduce IR, did not reach the levels of the NDC group (Figure a-d). The results of this test in both sexes of F1 offspring are similar to the parents of each group. F1 offspring of MgSO_4_ group females had a better effect on reducing GIR compared to males of MgSO_4_ group.

(Parents: NDC male: 13.3 ± .5 mg/dl vs DC male: 4.9 ± .76 mg/dl; NDC female: 21.0± 2.2 mg/dl vs DC female: 8.8 ± 1.0 mg/dl, Mg male: 11.1 ± .1 mg/dl; Mg female: 12.3 ± .63 mg/dl; insulin male: 8.1 ± .3 mg/dl; insulin female: 12.1± 1.09 mg/dl).

(F1 offspring: NDC male: 12.9 ± .9 mg/dl vs DC male: 2.3 ± .3 mg/dl; NDC female: 17.3± 1.0 mg/dl vs DC female: 3.3± .6 mg/dl, Mg male: 7.5 ± 1.3 mg/dl; Mg female: 9.0 ± .6 mg/dl; insulin male: 7.1 ± 1.3 mg/dl; insulin female: 10.5 ± 1.0 mg/dl).

### Effect of MgSO4 on HbA1c

**Figure 3:**
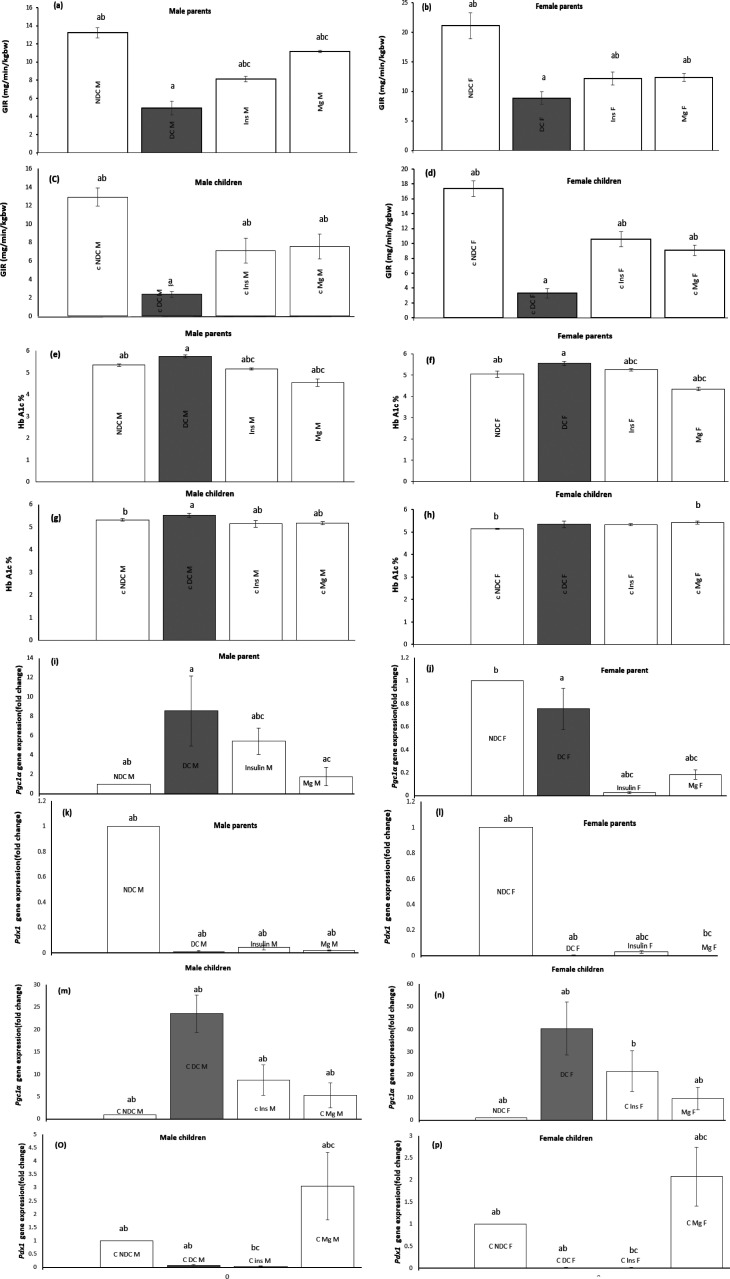
Comparison of glucose infusion rate (GIR) in male parents (a), female parents (b), male pups (c), female pups (d) and HbA1c in male parents (e), female parents (f), male pups (g), female pups (h) and Pgc1α gene expression in male parents (i), male pups (m), female parents (j), female pups (n) and Pdx1 gene expression in female parents (l), female pups (p), male parents (k), male pups (o) in non-diabetic control group (NDC) were fed standard diet, diabetic control group received high-fat diet and 35 mg/kg STZ (DC), diabetic animals were treated with 10 g/l MgSO_4_ by drinking water (Mg) and diabetic animals were treated with insulin (2.5 U/kg twice per day) (Insulin). STZ: Streptozotocin

The HbA1c measurements showed that the diabetes inducer caused a significant increase (*P*<0.01) in both males and females of the DC group compared to the NDC group. Additionally, our findings indicated a significant reduction (*P*<0.01) in HbA1c levels in both male and female participants in the groups receiving MgSO_4_ or insulin compared to the DC group. The reduction of HbA1c in both males and females in the MgSO_4_ group was significantly more effective compared to the insulin group (male: *P*<0.01; female: *P*<0.05, (Figure 3e-h). The results of HbA1c examined in F1 offspring showed that diabetes increases HbA1c in both male and female DC groups compared to male and female NDC groups. Our findings showed that administration of MgSO_4_ or insulin to parents reduced HbA1c in F1 offspring of these groups in both male and female genders.

(Parents: NDC male: 5.3 ± .06 mg/dl vs DC male: 5.7 ± .06 mg/dl; NDC female: 5.05± .7 mg/dl vs DC female: 5.5 ± .1 mg/dl, Mg male: 4.5 ± .1 mg/dl; Mg female: 4.3 ± .08 mg/dl; insulin male: 5.7 ± .04 mg/dl; insulin female: 5.2 ± .06 mg/dl).

(F1 offspring: NDC male: 5.3 ± .04 mg/dl vs DC male: 5.2 ± .08 mg/dl; NDC female: 5.1± .02 mg/dl vs DC female: 5.3 ± .1 mg/dl, Mg male: 5.1 ± .07 mg/dl; Mg female: 5.4 ± .07 mg/dl; insulin male: 5.1 ± .1 mg/dl; insulin female: 5.3 ± .0.4 mg/dl).

### Effect of MgSO4 on pancreatic genes expression (Pgc1α and Pdx1)

The expression of the *Pgc1α* and *Pdx1* genes was investigated in the pancreas. Induction of diabetes in both males and females significantly decreased *Pdx1* gene expression and increased *Pgc1α* gene expression compared to NDC groups (*P*<0.0001). Our findings showed that *Pdx1* gene expression in the MgSO_4_ or insulin-receiving group significantly increased in both sexes compared to the DC group, and *Pgc1α* gene expression decreased (*P*<0.0001). Similar changes in the gene expression of parents were also observed in their F1 offspring (Figure 3i-p).

### Effect of MgSO4 on serum insulin and glucagon levels

**Figure 4:**
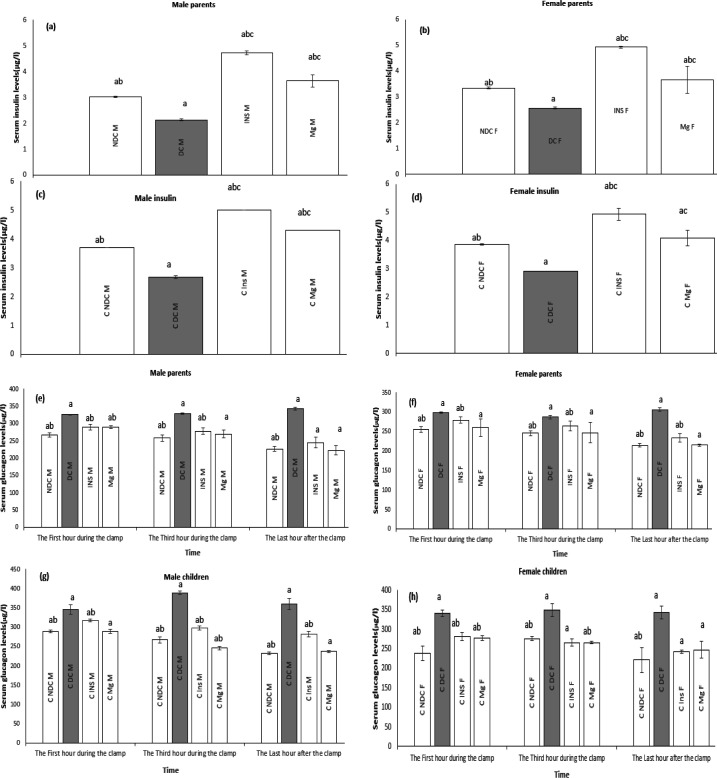
Comparison of serum insulin levels in male parents (a), female parents (b), male pups (c), female pups (d) and serum glucagon levels in male parents (e), female parents (f), male pups (g), female pups(h) in non-diabetic control group (NDC) were fed standard diet, diabetic control group received high-fat diet and 35 mg/kg STZ (DC), diabetic animals were treated with 10 g/l MgSO_4_ by drinking water (Mg) and diabetic animals were treated with insulin (2.5 U/kg twice per day) (Insulin). STZ: Streptozotocin

In both male and female parents and their F1 offspring, the insulin levels of the DC group were significantly reduced (*P*<0.0001) compared to the NDC group. In the groups receiving MgSO_4_ or insulin, insulin levels increased significantly (*P*<0.0001) compared to the DC group, with a higher increase observed in the insulin group compared to the MgSO_4_ group (Figure 4a-d) (*P*<0.001). During the first and third hours, glucagon levels in both sexes of parents and their F1 offspring in the DC group showed a significant increase (*P*<0.0001) compared to the NDC group. Glucagon levels were decreased in the MgSO_4_ or insulin groups compared to the DC group (*P*<0.001). In the last hour of the treatment groups, the decrease in glucagon level was significant compared to the previous two time points (Figure 4e-h) (*P*<0.0001). In the group receiving MgSO_4_, the decrease in glucagon levels in the last hour was higher compared to the insulin group in both sexes, with significance observed in the female parents’ group (*P*<0.01) and the male F1 offspring group (*P*<0.001).

### Effect of MgSO4 on HOMA-IR and TyG index

**Figure 5:**
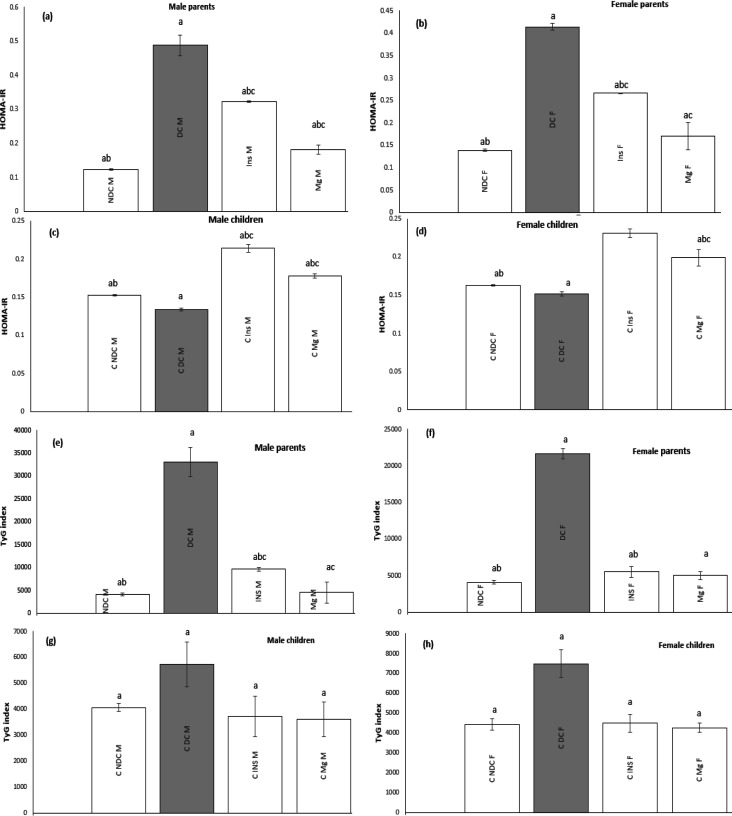
Comparison of HOMA-IR in male parents (a), female parents (b), male pups (c), female pups (d) and TyG index in male parents (e), female parents (f), male pups (g), female pups (h) in non-diabetic control group (NDC) were fed standard diet, diabetic control group received high-fat diet and 35 mg/kg STZ (DC), diabetic animals were treated with 10 g/l MgSO_4_ by drinking water (Mg) and diabetic animals were treated with insulin (2.5 U/kg twice per day) (Insulin). STZ: Streptozotocin

In both male and female parents and their F1 offspring, HOMA-IR (Figure 5a-d) and TyG (Figure 5e-h) indices of the DC group were significantly increased (*P*<0.0001) compared to the NDC group. In the groups receiving MgSO_4_ or insulin, the levels of HOMA-IR and TyG indices decreased significantly (*P*<0.0001) compared to the DC group, with a greater decrease observed in the MgSO_4_ group compared to the insulin group (*P*<0.001). The changes in HOMA-IR and TyG results in F1 offspring aligned with the parents’. Both factors showed reliable positive results in both F1 offspring of the MgSO_4_ and insulin groups compared to the DC group.

## Discussion

This study investigated the effect of MgSO_4_ on improving IR in T2DM parents and their F1 offspring after HFD feeding. In addition, the HEC was used as the gold standard method to assess IR in conscious rats. The following tests were also utilized in the present study: 1. BGL and body weight to monitor the improvement of glycemic indices in diabetes 2. ITT, HbA1c, HOMA-IR, TyG index, and glucagon levels to quantify IR 3. Expression of *Pgc1α* and *Pdx1* genes and insulin levels to evaluate the survival rate and regeneration of pancreatic β-cells. The results of this study indicated that the consumption of HFD by T2DM parents could result in elevated BGL, impaired ITT, obesity, and IR in their F1 offspring compared to F1 offspring of normal parents. Furthermore, the administration of MgSO_4_ or insulin was found to improve BGL, ITT, body weight, IR, HbA1c, and the expression of *Pgc1α* and *Pdx1* genes in diabetic parents. While also increasing insulin levels and decreasing glucagon levels. Changes in glucagon levels were used as a measure of IR. These findings demonstrated that all IR indices mentioned above improved in the MgSO_4_ group (Table 3). 

Our results showed that the injection of STZ and administration of HFD led to increased blood glucose levels in both male and female rats. Also, the blood glucose levels in the F1 offspring of diabetic rats were significantly higher compared to both sexes of the MgSO_4_-treated group. Obesity is a significant risk factor for diabetes, with most individuals being overweight or obese. Obesity results in increased fat storage in adipose tissues, which can become overwhelmed if energy intake exceeds a certain point. Excess energy leads to increased lipolysis in adipose tissues, releasing FFAs into the bloodstream, where they accumulate. This elevation in circulating FFAs contributes to hepatic and muscle IR and interferes with pancreatic β-cell insulin secretion(30). While the exact mechanism by which lipids induce IR is still debated, the prevailing theory suggests that intracellular lipid metabolites disrupt insulin signaling, leading to decreased glucose uptake in skeletal muscles and adipocytes, increased hepatic glucose production, and impaired pancreatic insulin synthesis, ultimately resulting in hyperglycemia (31).

Diabetes and obesity (HFD) are closely related (32). Consuming too many calories increases body weight and fat mass, causing body cells to come into contact with a significant amount of fat(33, 34). Free fatty acids (FFA) are known to increase peripheral IR. However, FFAs also support basal insulin secretion and enhance glucose-stimulated insulin secretion. As a result, diabetic patients can compensate for FFA-mediated IR with increased FFA-mediated insulin secretion. Some patients, however, cannot do this and eventually develop T2DM. Elevated plasma levels of FFAs are a significant cause of IR in skeletal muscle and liver. They may also play a role in the development of IR in other insulin target cells(35). This means that fat covers the insulin receptors on the surface of insulin-target tissue cells, preventing insulin from binding to its receptor and causing the body’s cells to respond less to insulin(36, 37). This disruption in the response to insulin leads to disrupted glucose metabolism and dysfunction of β-cells, resulting in increased blood glucose or hyperglycemia (38, 39). Increased blood glucose levels contribute to IR, raising BGL by reducing insulin production and interfering with glucose absorption (40). IR also helps promote increased glucagon production, which in turn raises BGL. Glucagon exacerbates hyperglycemia by stimulating glycogen breakdown and gluconeogenesis (41). HbA1C reflects average blood glucose control over time, influenced by the interplay of these factors (42).

The fact that BGL in diabetic rats increased significantly after receiving HFD is consistent with the current study’s findings. Available data suggested that excessive fat deposition in non-adipose tissues such as skeletal muscle, the heart, the liver, and the pancreas may lead to the abnormal release of fatty acids and inflammatory substances, which could impair the function of these tissues and result in IR. Fat infiltration into the pancreas, which negatively affects systemic metabolism, is believed to contribute to the pathophysiology of T2DM (43). Studies have shown that excess pancreatic fat can decrease insulin secretion and accelerate apoptosis in pancreatic β-cells through various processes, including the release of local FFAs, proinflammatory factors, vasoactive substances, oxidative stress, and the accumulation of triglyceride metabolites (44, 45). This aligns with the results of serum insulin levels obtained in our study. Previous research has also indicated that obese individuals with T2DM exabit a weakened first-phase insulin response (46), consistent with our findings from serum insulin measurements. Our study compared serum insulin levels between the DC and NDC groups, revealing a significant difference. Another study found that patients with pancreatic fatty infiltration had lower fasting and postprandial BGL and impaired β-cell function compared to healthy individuals, suggesting a potential link between β-cell dysfunction and glucose metabolism disorders (47). Previous research has shown that the impact of dietary fat content on the risk of diabetes differs between genders, with females being less susceptible to diabetes. This gender disparity may be attributed to the presence of sex steroid hormones, such as estrogens in females (48).

Furthermore, the present study’s findings showed that receiving HFD and inducing diabetes disrupted pancreatic tissue function, glucose level, and ITT, leading to increased IR, as confirmed by the GIR obtained from the clamp. The changes related to these factors in the F1 offspring of both sexes were almost in line with those observed in the parents of both sexes. As mentioned in the results section, some changes in the female sex of F1 offspring showed a more significant improvement than the male F1 offspring. Therefore, by receiving an HFD and inducing diabetes, various cascade reactions such as oxidative stress, endoplasmic reticulum stress, and inflammatory response may have been activated and aggravated β-cell dysfunction. The pancreas is a crucial organ for the metabolism of fat and glucose. However, research on pancreatic fat content and its association with IR is still rare and has shown conflicting results. A study reported a correlation between pancreatic fat with HOMA-IR values and glucose levels (49). The current study’s findings supported other studies by demonstrating a favorable link between HFD and HOMA-IR, glucose, and IR levels. These results suggest that the HFD-induced increase in fat content can impact pancreatic IR. Hypoglycemia stimulates glucagon secretion in healthy individuals, but this counterregulatory mechanism is impaired in diabetes, leading to continued stimulation of glucagon secretion by hypoglycemia. Glucagon secretion is controlled by paracrine factors released by β and δ cells in response to glucose levels. These factors suppress insulin secretion from β-cells and glucagon through acting on α-cells. Insulin also inhibits glucagon secretion by an indirect (paracrine) mechanism mediated by stimulation of somatostatin release (50). 

This study showed that administering MgSO_4_ to diabetic animals increased insulin levels in both sexes of diabetic parents and F1 offspring compared to the DC group. This increase in insulin levels may have also caused a decrease in glucagon levels in this group, followed by a decrease in IR compared to the DC group. The importance of fatty acids during pregnancy cannot be overstated. The role of fatty acids in pregnancy is crucial for growth and development. Fatty acids are supplied to the fetus during pregnancy based on the mother’s nutrition. In addition to crossing the placenta during pregnancy, fatty acids also play a significant role in postnatal development through breast milk (51). Cell structure and function can be affected both short- and long-term by changes in the metabolic environment caused by inadequate or excessive maternal nutrition (52). Consequently, structural and functional changes in metabolism are caused by the imbalance in fatty acid intake during fetal growth. Changes in the metabolic environment during development may result in the later development of chronic disorders (53). The study’s findings have significant implications for our understanding of the effects of MgSO_4_ on IR and glucagon levels in diabetic animals. The results suggest that MgSO_4 _may be a helpful therapeutic agent for managing IR and improving glucose regulation in diabetic animals. Additionally, the study highlights the importance of adequate maternal nutrition during pregnancy, as changes in the metabolic environment during fetal development can have long-term consequences for metabolic health.

It has been reported that there are gender disparities in the prevalence of T2DM shows gender disparities with a lower incidence in women. In postmenopausal women, decreased estrogen accelerates the development of IR and T2DM because estrogen helps lower glucose levels. Estrogen protects female adipose tissue against fat hypertrophy, oxidative stress, and IR (54, 55). Trace elements, such as Mg^+2^, are also essential during intrauterine life when fetal growth occurs, and insufficient tissue Mg^+2^ concentrations can have significant adverse effects on fetal birth weight (56). Mg^+2^ deficiency is more common in women than in men. This may be due to estrogen stimulation of Mg^+2^ by tissues, and therefore hormonal rhythms in women may affect and modulate Mg^+2^ status (54, 57). A previous study showed that pancreatic β-cell dysfunction, IR, and T2DM are associated with Mg^+2^ deficiency (58). It is also consistent with the IR results obtained from this study. IR was lower in female than male animals. Therefore, the protective effects of estrogen on lipid distribution and oxidative stress (59, 60) and its role in maintaining healthy magnesium levels may contribute to the reduced incidence of T2DM in female animals compared to male animals.

Since Mg^+2^ regulates electrical activity and insulin secretion in pancreatic β-cells, intracellular Mg^+2^ concentration plays a crucial role in the phosphorylation of insulin receptors and other downstream signal kinases in target cells. Low levels of Mg^+2^ can lead to defective tyrosine kinase activity, impairment of insulin action post-receptor, altered cellular glucose transport, reduced cellular glucose utilization, and promotion of peripheral IR in T2DM. Low levels of Mg^+2^ also cause chronic systemic inflammation, further promoting IR. Individuals with T2DM may be caught in a vicious cycle where low Mg^+2^ levels increase IR, and IR causes low Mg^+2^ levels(12). Although the exact mechanism is unknown, studies have suggested that Mg^+2^ deficiency during pregnancy may contribute to fetal IR (61, 62). One of the signs of IR is ITT dysfunction, and the current study’s findings indicate that MgSO_4 _treatment had a greater impact on this indicator in both parents and their F1 offspring than insulin therapy. Another indicator of IR is GIR, and our results demonstrated that administration of MgSO_4_ or insulin increased GIR in both parents and their male and female F1 offspring. It was also observed that the increase in GIR was more significant in female and male animals. HbA1c a marker of IR, increased in both parents following HFD consumption. MgSO_4_ or insulin therapy was able to reduce HbA1c levels in both parents and their male and female F1 offspring.

Based on our findings, we concluded that the F1 offspring of diabetic rats whose parents were treated may exhibit positive results in the measured factors. While we do not know the exact cause of diabetes in the F1 offspring of diabetic rats whose parents were treated, it is likely that a combination of factors could play a role. However, it highlights the importance of considering the potential franchising effects of treatments on health outcomes and underscores the need for further research in this area.

**Figure 6:**
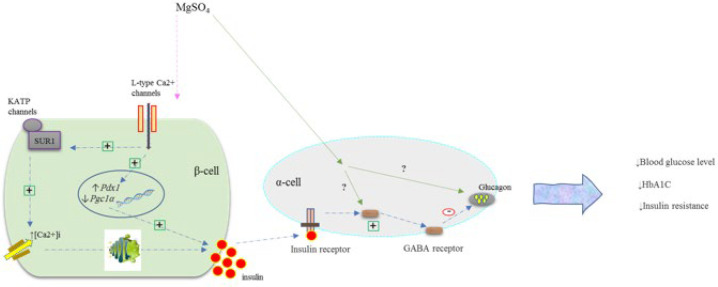
Communication pathways of the effect of MgSO_4_ on the pancreas in diabetic rats

This study also showed that the administration of MgSO_4_ increased the expression of the *Pdx1* gene and decreased the expression of the *Pgc1α* gene. Therefore, MgSO_4_ administration appears to improve IR through the insulin signal transduction pathway. The results also showed that MgSO_4_ treatment can increase serum insulin levels and decrease glucagon levels. Figure 6 of the current research illustrates the potential mechanism of MgSO_4_ to improve pancreatic IR.

Changes in factors such as HOMA-IR, TyG index, BGL, ITT, HbA1c, glucagon levels, IR, and GIR suggest that treatment with MgSO_4_ and insulin may improve glucose metabolism, improve insulin response, and help reduce the risk of T2DM in diabetic patients and future generations. In addition, changes in gene expression, especially in *Pgc1α* and *Pdx1*, indicate a positive effect on pancreatic β-cells’ survival and ability to regenerate. These changes could be of great importance for preventive and restorative treatments in T2DM and metabolic disorders. 

**Table 1:** Nutritional composition and ingredients of the high-fat diet in F1 offspring of diabetic rats

Ingredients	Diet (g/kg)
Powdered NPD	365
Lard	310
Casein	250
Cholesterol	10
Vitamin and mineral mix	60
DL-Methionine	3
Yeast powder	1
Sodium chloride	1

**Table 2:** Primers for quantitative real-time PCR analysis of gene expression in rat pancreas

Gene	Reveres primer	Forward primer	Ref.
Beta-actin	CTGACCCATACCCACCATCAC	ACAACCTTCTTGCAGCTCCTC	designed with NCBI's Primer-BLAST
*Pdx1*	TGTAGGCTGTACGGGTCCTC	CCCGAATGGAACCGAGACTG	designed with NCBI's Primer-BLAST
*Pgc1α*	CAAAGAGGCTGGTCCTCACC	TGACTGGCGTCATTCAGGAG	designed with NCBI's Primer-BLAST

a: Significant difference between DC and NDC groups, body weight (*P*<0.001), BG (*P*<0.0001), b: Significant difference between NDC and Treatment groups (*P*<0.001), c: Significant difference between DC and treatment groups (*P*<0.001), d: Significant difference between Insulin and Mg groups (*P*<0.001).

a: Significant difference between DC and NDC groups (*P*<0.0001). b: Significant difference between NDC and Treatment groups (*P*<0.01). c: Significant difference between DC and Treatment groups (*P*<0.0001). d: Significant difference between Insulin and Mg groups (*P*<0.01).

a: Significant difference between DC and NDC groups (*P*<0.0001), b: Significant difference between NDC and Treatment groups (*P*<0.001), c: Significant difference between DC and Treatment groups for GIR (*P*<0.001), d: Significant difference between Insulin and Mg groups, GIR (*P*<0.01) and HbA1c (*P*<0.05).

a: Significant difference between DC and NDC groups (*P*<0.001), b: Significant difference between NDC and Treatment groups (*P*<0.001), c: Significant difference between DC and Treatment groups (*P*<0.001), d: Significant difference between Insulin and Mg groups (*P*<0.01).

a: Significant difference between DC and NDC groups (*P*<0.001), b: Significant difference between NDC and Treatment groups (P<0.001), c: Significant difference between DC and Treatment groups (*P*<0.001), d: Significant difference between Insulin and Mg groups (*P*<0.01).

**Table 3:** Summary of findings: This table compares various physiological and biochemical factors between F1 offspring and parents, with a breakdown by gender (male/female). Factors such as glucose levels, hormones, gene expression, and insulin sensitivity are compared. The symbols "↑" indicate an increase, "↓" a decrease, and "N" signifies no change when comparing different groups. These data explore metabolic and physiological changes in F1 offspring and parents

F1 Offspring								Parents								Factors
Male				Female				Male				Female				Gender
Mg	Insulin	DC	NDC	Mg	Insulin	DC	NDC	Mg	Insulin	DC	NDC	Mg	Insulin	DC	NDC	Index
↑	↑	↓	N	↑	↑	↓	N	↑	↑	↓↓	N	↑	↑	↓↓	N	GIR
↓	↓	↑	N	↓	↓	↑	N	↓	↓	↑	N	↓	↓	↑	N	HbA1c
↓	↓	↑	N	↓	↓	↑	N	↓	↓	↑	N	↓	↓	↑	N	HOMA-IR, TyG index
↓↓	↓	↑	N	↓	↓↓	↑	N	↓↓	↓	↑	N	↓↓	↓	↑	N	*Pgc1α*gene expression
↑	↑	↓	N	↑	↑	↓	N	↑↑	↑	↓	N	↑↑	↑	↓	N	*Pdx1* gene expression
↑	↑	↓	N	↑	↑	↓	N	↑	↑	↓	N	↑	↑	↓	N	Insulin level
↓	↓	↑	N	↓	↓	↑	N	↓↓	↓	↑	N	↓↓	↓	↑	N	Glucagon levels
↓	↓	↑	N	↓	↓	↑	N	↓	↓	↑	N	↓	↓	↑	N	Blood glucose level
↑	↑	↓	N	↑	↑	↓	N	↓	↓	↓	N	↓	↓	↓	N	body weight
↓	↓	↑	N	↓	↓	↑	N	↓	↓	↑	N	↓	↓	↑	N	(AUC) in ITT

## Conclusion

According to the current research results, consuming MgSO_4_ positively improves glycemic indices and IR in diabetic rats treated with STZ. MgSO_4_ consumption likely improved blood glucose levels and reduced IR in the study due to its impact on pancreatic β-cell survival, insulin levels, increasing the capacity of insulin to bind to its receptors, and decreasing glucagon secretion by pancreatic α-cells. Given that the majority of relevant factors were examined in this study and the results were promising, it seems that these findings can be used to assess the clinical potential of MgSO_4_ in treating diabetes and metabolic disorders. These results highlight the importance of the proposed therapeutic approach and suggest it could offer a novel strategy for managing metabolic diseases. However, to confirm and extend these findings, further extensive and long-term studies in humans are necessary to fully evaluate the efficacy and safety of this treatment in diverse populations.

## Data Availability

All the data are given in the article.

## Declaration

We did not use any AI tools or technologies in preparing this manuscript.

## Study Limitations

Although this study has examined a wide range of data and conducted various analyses, there are still some limitations that should be considered:

One weakness of the study was the lack of radioactive glucose in IR tissue analysis. Therefore, it is recommended that this factor be included in future studies. The study used diabetic rat models, which, although similar to humans, may not fully replicate the complexity of human metabolic diseases. Future research should explore other animal models or even human clinical studies to validate the findings and provide more reliable results. The study did not assess the long-term side effects or the development of tolerance to MgSO_4_. A longer study is needed to evaluate the sustained impact and safety of the treatment. While the study focused on the effects of MgSO_4_ on *Pdx1*and *Pgc1α* genes, IR is influenced by numerous genetic and environmental factors. Future studies should also examine the impact of MgSO_4 _on other genes and molecular pathways related to IR. Radioactive glucose is recommended to assess MgSO_4_’s effects in future studies accurately. Future research should investigate the combination of MgSO_4_ with other treatments, such as antidiabetic drugs, dietary changes, or exercise programs. This approach could lead to more effective therapeutic strategies for managing diabetes and IR.
